# Pico-Dispensed Zinc Oxide Nanoparticles for Actuation of Microcantilevers: A Precise Deposition Approach

**DOI:** 10.3390/s25123689

**Published:** 2025-06-12

**Authors:** Paweł Janus, Anna Katarzyna Piotrowska, Piotr Prokaryn, Andrzej Sierakowski, Jan Prokaryn, Rafał Dobrowolski

**Affiliations:** Łukasiewicz Research Network–Institute of Microelectronics and Photonics, 02-668 Warsaw, Poland; anna.piotrowska2@imif.lukasiewicz.gov.pl (A.K.P.);

**Keywords:** zinc oxide nanoparticles, self-actuation, AFM microcantilevers, electromechanical response, pico-dispensing technology

## Abstract

This paper presents a cost-effective and versatile pico-dispensing technique as an efficient and straightforward approach for depositing zinc oxide nanoparticle (ZnO—NP) thin films on micromechanical devices (MEMS). Due to its piezoelectric properties, bulk ZnO is commonly used as a material for micro-/nanocantilever actuation. The pico-dispensing process provides precise control over the deposition, allowing uniform and localized application of ZnO—NP on microcantilevers. Compared to traditional ZnO deposition techniques (e.g., sputtering or sol–gel), pico-dispensing of ZnO—NP offers advantages in simplicity, reduced material waste, and significantly lower costs. Furthermore, it is easy to tailor the composition and properties by incorporating nanoparticles of other materials. Experimental results demonstrate that ZnO—NP thin films deposited via pico-dispensing enable actuation with amplitudes of several nanometers and bandwidths up to 250 kHz, making them potentially suitable for actuation of micromechanical devices such as in dynamic AFM modes.

## 1. Introduction

Atomic force microscopy (AFM) relies on precise control and actuation of cantilevers to achieve high-resolution imaging and material characterization at the nanoscale. Traditional actuation methods, such as piezoelectric, magnetic, or thermal, face challenges related to cost, complexity, and integration of miniaturized systems [1,2]. Recent progress in nanomaterials has opened new pathways for efficient actuation. Among these, the use of pico-dispensed zinc oxide (ZnO) nanoparticles may offer a novel and promising approach to actuate AFM cantilevers for operation in dynamic modes. The wurtzite structure provides piezoelectric properties of bulk zinc oxide. Hence, zinc oxide converts electrical energy efficiently into mechanical motion. The most popular methods for manufacturing ZnO layers are sputtering, chemical vapour deposition (CVD), and sol–gel. These provide high-quality, dense films with well-defined crystallography. However, these methods do not allow the local deposition of ZnO structures. Subsequent photolithography processes and removal of the redundant layer are necessary. Piezoelectric properties of ZnO nanoparticle layers were already utilized in piezoelectric nanogenerators, sensors, and energy-harvesting devices [3]. To assess the feasibility of actuation using pico-dispensed ZnO nanoparticles as a proof-of-concept platform, building on our previous experience in AFM cantilever design, we selected the dynamic mode atomic force microscopy as the test application.

The dynamic mode of AFM [4,5], such as the tapping mode [6] or the non-contact mode [7], relies on the vibration of a cantilever near its resonant frequency. This mode provides advantages over static modes by reducing sample damage and allowing measurements of delicate or soft materials. An external actuator induces cantilever oscillations with the desired frequency and the amplitude of nanometers [8].

ZnO nanoparticles, as an integrated actuator, offer a simplified and flexible method that avoids complex processing steps, such as sputtering and patterning. This makes it particularly suitable for applications where conventional actuators cannot be integrated, such as post-processing scenarios involving already-functionalized sensors with sensitive biochemical layers.

Using the AFM measurement techniques, a general characterization of the structure and related properties of the ZnO—NP thin film was obtained. However, the possibility of an electrostatic contribution to the ZnO—NP sample deformation, in addition to the piezoelectric effect, could not be excluded. The significance of this factor for AFM measurements has been extensively discussed in [9,10]. Accordingly, an effective electromechanical coefficient associated with the ZnO—NP sample deformation was defined and evaluated based on the AFM tip deflection as a function of the applied bias voltage.

## 2. Materials and Methods

The ZnO—NP layer was locally deposited from a 2.5% solution of crystalline ZnO in 2-propanol (zinc oxide nanoparticle ink, SIGMA-ALDRICH, Darmstadt, Germany [11]). Particles sized from 10 nm to 15 nm support layering a continuous film. Before applying to the cantilever, the solution was filtered through a 0.45 µm-PTFE (polytetrafluoroethylene) filter and placed in an ultrasound bath for 10 min to break up the agglomerates.

**Figure 1:**
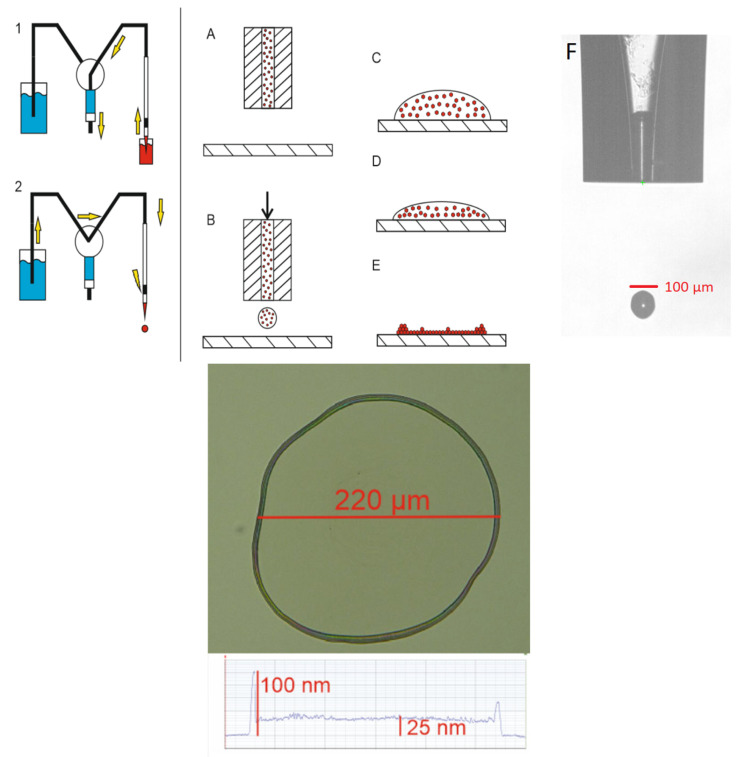
Diagram of the dispensing process with yellow arrows indicating flow of the liquids, photo of a drop formed by the nozzle, and a profile of a drop on the surface.

The deposition involves a pico-dispenser (Scenion, sciFLEXARRAYER S12, Berlin, Germany) and glass nozzles equipped with piezo actuators. This allows for accurate and repeatable placement of picolitre volumes of liquid droplets. Firstly, by creating negative pressure in the nozzle through movement of the syringe pump, the sample is aspirated into the nozzle (Figure 1(1)). After the pump stops, the system is prepared for dispensing liquid using a piezoelectric device mounted on the corpus of the nozzle (Figure 1(2)). Adjusting the actuating signal to the parameters of the substance results in a stable and reproducible droplet (Figure 1(F)). After centralizing the nozzle at 500 µm above a chosen substrate (Figure 1(A)), the actuating signal is applied, which produces hypertension ejecting droplets of substance (Figure 1(B)). NPs in the deposited material are spread evenly (Figure 1(C)). The drying process leads to NPs slowly drifting towards edges, where they form a ring-shaped structure (Figure 1(D,E)). This is called ‘the coffee-ring effect’ [12]. The flat-disc portion of deposited material has a thickness of 20 nm, whereas the ring structure is 100 nm to 120 nm thick. Measurements were conducted using the Stylus Profiler System (Bruker, DektakXT, Tucson, AZ, USA) with an error of 0.5 nm.

**Figure 2:**
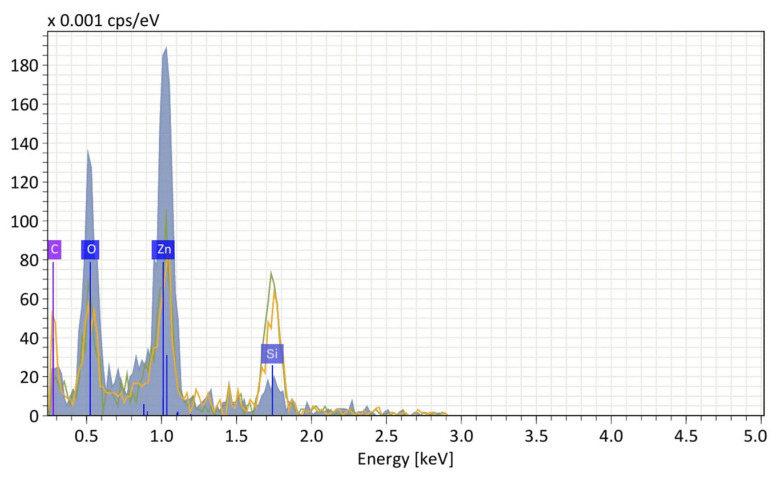
EDS pattern of the ZnO—NP layer dispensed on the Si substrate.

The tests were intended to assess the composition of the layer after the solvent evaporation was performed [13]. The ZnO—NP spots were deposited onto the silicon substrate [14,15]. SEM/EDS (energy dispersive spectroscopy) and AFM were used to evaluate the material’s quality and surface morphology (an average root mean square (RMS) roughness of the surface before deposition—0.2–0.4 nm). EDS measurements were conducted using EDAX Elite T EDS with FEI Nova™ NanoSEM 630 (EDS—AMETEK GmbH, Weiterstadt, Germany; NanoSEM—FEI Europe B.V., Eindhoven, The Netherlands) The obtained data suggest a material primarily composed of zinc oxide (Zn-1 keV, O-0.5 keV), with possible surface contamination or a carbonaceous matrix at 0.2 keV. The energy peak equal to 1.7 keV is a contribution of the silicon substrate. Figure 2 shows the energy pattern in the EDS spectrum.

**Figure 3:**
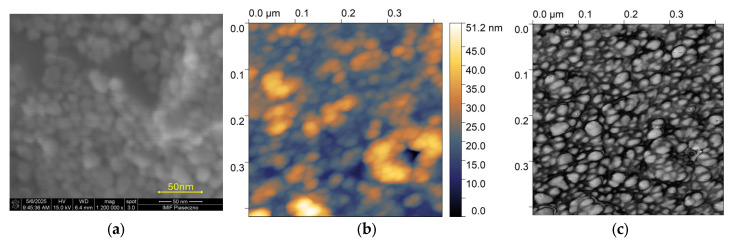
Topography image provided by (**a**) SEM and (**b**) AFM; (**c**) phase-shift diagram of the single-layer deposited ZnO nanoparticles, showing the surface morphology; the scan field is 400 nm × 400 nm and the median of grain size is 20.46 nm.

Figure 3a,b illustrates the topography and the phase shift in the tested surface. The obtained results were utilized to ascertain the grain size of deposited particles.

**Figure 4:**
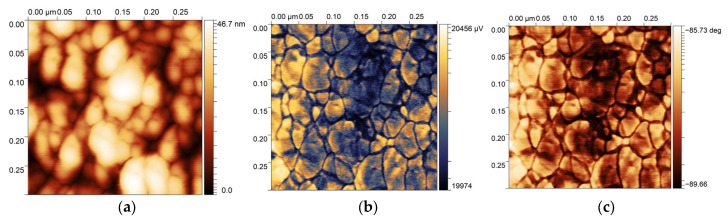
AFM scans of the multilayer ZnO—NP sample surface (the scan field is 300 nm × 300 nm): (**a**) topography image; (**b**) amplitude image; and (**c**) phase-shift image.

The multilayer ZnO—NP sample deposited on the silicon substrate covered with metal (20 nm Au, roughness 0.2–0.4 nm) was tested using the piezoresponse force microscopy (PFM) technique (Nanosurf FlexAFM C3000 equipment, Nanosurf AG Liestal, Switzerland) [16,17] (Figure 4). The conductive AFM probe (NanoWorld CONTPt, Neuchâtel, Switzerland) was brought into contact with the sample. The AC excitation voltage (6 V, 60 kHz) was applied between the tip and the sample. To reduce the impact of humidity variations, the measurements were performed atthe controllable lab conditions, i.e., at stabilized relative humidity of 30% and temperature of 22 ± 1 °C.

**Figure 5:**
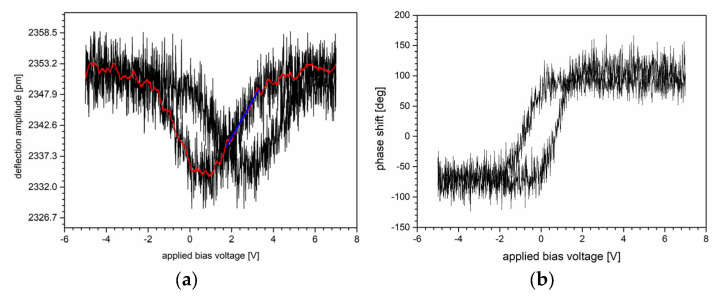
Electromechanical response hysteresis loop of ZnO—NP layer (**a**) amplitude vs. applied voltage and (**b**) phase vs. applied voltage obtained by switching spectroscopy PFM [17]. Here, the black curves correspond to the experimental data, the red curve was obtained by applying the Savitzky-Golay algorithm, and the straight blue line is the tangent line referring to the linear relationship between the tip deflection amplitude and the applied bias voltage.

Figure 5 shows two characteristics of the ZnO—NP sample obtained from PFM spectroscopic measurements. Two graphs clearly show the relationship between the applied bias voltage and the strain appearance in the analyzed structure. The drop near 0 V may correspond to domain instability, suggesting that the material does not expand or contract uniformly. Furthermore, the hysteresis reflects the nonlinear, electromechanical response of the sample. However, this effect requires extensive studies to relate it to ZnO—NP piezoelectric properties and to skip other effects, such as electrostriction.

Based on the approach reported in [18], we estimated the effective electromechanical coefficient as a rate of the tip deflection and the applied bias voltage. The calculations were performed for the data range corresponding to the linear relationship between the tip deflection and the applied bias voltage marked by the blue straight line in Figure 5a. The estimated value amounted to 5.2 pm/V and refers to the slope of the tangent line.

**Figure 6:**
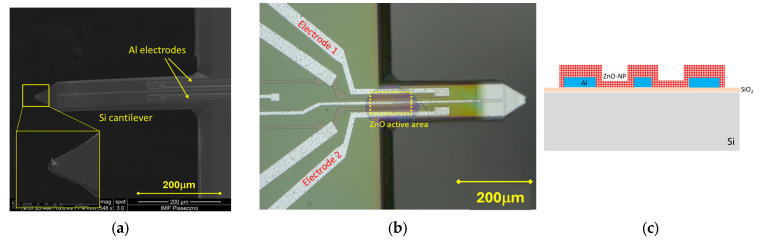
(**a**) SEM image of the AFM cantilever used in the experiment; (**b**) cantilever with the dispensed ZnO—NP marked by the yellow, dashed-line rectangle; (**c**) cross-section of the ZnO—NP active area in (**b**).

The ZnO—NP layer pico-dispensing was performed on the AFM cantilevers with a geometry applicable for dynamic modes (k ~3 N/m, frequency ~100 kHz, Q ~300). The probes, manufactured at the Institute of Microelectronics and Photonics (IMiF, Piaseczno, Poland), are equipped with planar aluminium electrodes and a 10 nm sharp silicon tip (Figure 6). Additionally, the probes are integrated with a piezoresistive deflection sensor, which was disconnected for the current experiment. The described experiment used a configuration that did not require an upper electrode. Despite the disadvantage of such a solution, which is a less well-defined capacitor, the main advantage is simplified manufacturing technology.

## 3. Measurements and Evaluation

### 3.1. The Resonance Parameters Determined by the Laser Doppler Vibrometry Technique

To assess the mechanical properties (the deflection amplitude and the related resonance frequency) of the AFM microcantilevers, a microsystem analyser (MSA-500, Polytec, Waldbronn, Germany) was employed. Polytec MSA-500 is a versatile and functional tool for MEMS design and evaluation. It operates based on the laser Doppler vibrometry technique, providing precise and reliable measurements.

The measurements were performed before and after ZnO deposition on four tested probes. The resonance effect was induced through the probes’ Brownian motion (the self-excited, harmonic vibrations of the AFM probes [19]) and by applying voltage stimulation (V_gen_ = 10 V) from the integrated Polytec MSA-500 generator.

**Figure 7:**
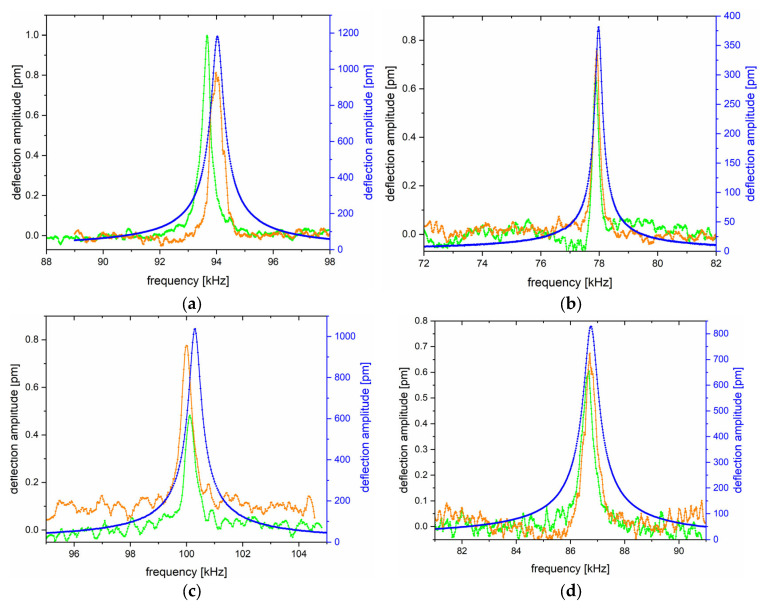
The resonance effect is depicted as a function of the deflection vs. the frequency, measured for the probes: (**a**) 1, (**b**) 4, (**c**) 5, and (**d**) 6. The left-handed, black *y*-axis refers to the deflection amplitude of Brownian motions, and the right-handed, blue *y*-axis refers to the amplitude of deflection induced by the generator. The green resonance curve refers to the Brownian motion of the AFM microcantilevers before ZnO deposition; the orange resonance curve refers to the Brownian motions of the AFM microcantilevers with the ZnO component; and the blue resonance curve refers to the AFM microcantilevers’ oscillations stimulated by the external generator.

Figure 7 shows four graphs with three resonance curves for each microcantilever separately. The green resonance curve refers to the Brownian motion of the microcantilever beams without the deposited ZnO—NP, the orange curve refers to the Brownian motion of the microcantilever beams with the deposited ZnO—NP, and the blue curve was measured on the probes after the ZnO—NP deposition with applied voltage stimulation from the generator. Table 1 presents the values of the resonance frequencies and the related deflection maxima for each probe before and after ZnO—NP deposition.

The resonance frequency changes after ZnO—NP depositions are negligible. The resonance frequency increased for probes 1 and 6 and decreased for probes 4 and 5. However, these changes are negligibly small (a few tens or hundreds of Hz). By applying the external voltage, the magnitude of deflection amplitude was significantly increased, reaching up to 1 nm (probes 1 and 6), which was considered to be a sufficient value for further experiments in the AFM system.

### 3.2. AFM Evaluation

**Figure 8:**
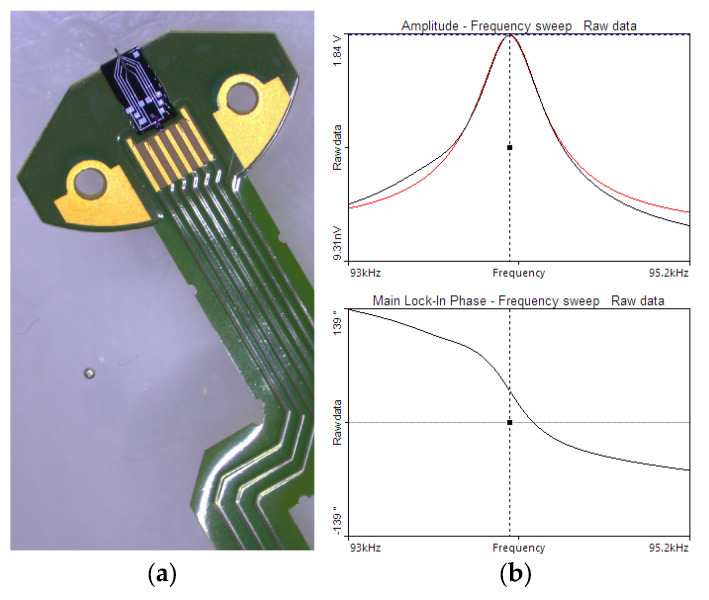
(**a**) The probe assembled on the dedicated flex-PCB; (**b**) the resonance curve measured inside the AFM system with the ZnO—NP actuation (black—measured, red—fitted).

To evaluate the performance of cantilevers with the integrated ZnO—NP actuation in dynamic mode, several scans were conducted using a commercial Nanosurf Flex-AFM system. The calibration gratings (Raith Chessy Test Specimen [20]) with a nominal height of 100 nm and periodicity of 1 µm were used to evaluate the cantilevers’ imaging accuracy and dynamic response. The scans were conducted under laboratory conditions (21 ± 1 °C, 30 ± 1%RH). Furthermore, the driving voltage equal to 10 V was applied to the ZnO—NP actuation spot, resulting in the AFM deflection of the tip equal to 1 nm. In the experiments, the cantilever installed in the AFM system was driven by an external generator controlled by FlexAFM electronics. Readout was performed using a standard OBD (optical beam deflection) system of the AFM setup. Figure 8b shows the resonance curves recorded in the AFM microscope system.

**Figure 9:**
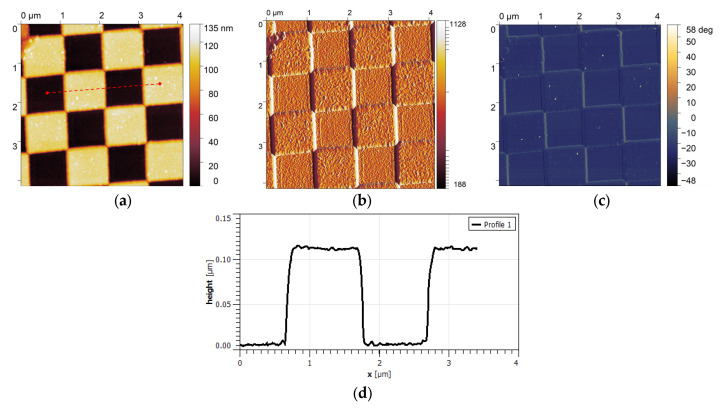
(**a**) Topography image of the test gratings, showing uniform step heights and transitions; (**b**) amplitude map that visualizes the stability of the cantilever oscillations during scanning (in pm). (**c**) phase-shift map, highlighting the mechanical response of the sample surface, confirming the cantilever’s sensitivity to material properties; and (**d**) cross-section of the test grating alongside the red, dashed line scan marked in (**a**).

Figure 9 shows the results of the measurements performed in the non-contact mode in the scan area of 4 μm × 4 μm, with the scanning speed equal to 1 line/s. Figure 9a presents the topography image of the test gratings with the uniform step heights and transitions. Figure 9b,c show the amplitude and the phase-shift map, visualizing the stability of the cantilever oscillations during scanning.

The image statistical analysis provides an average scanned square element size of the sample of 1.07 µm and a depth of 105 nm, which is consistent with the nominal values.

## 4. Conclusions

This study explores the application of zinc oxide (ZnO) nanoparticle layers, deposited at the fixed end of silicon AFM cantilevers, as a potential actuation mechanism for dynamic mode operation. The electromechanical response of the ZnO—NP structure was confirmed through PFM measurements. However, extracting the detailed piezoelectric properties of the material requires further investigation, which is planned for future work. The possibility of leveraging ZnO—NP layers to enable simplified self-actuated and self-sensing microcantilevers remains an area of ongoing interest.

It is important to note that the actuation amplitude demonstrated here (~1 nm) remains below levels typically required for dynamic AFM operation. The present work should therefore be regarded as a proof-of-concept platform to assess the feasibility of pico-dispensed ZnO—NP actuation, rather than a fully optimized device implementation. Further optimization—including advanced electrode configurations, control of nanoparticle layer thickness, and exploration of multilayer structures—will be necessary to achieve practical performance levels. In addition to AFM applications, this actuation approach may prove useful in selected MEMS, such as low-force switches or microbridge resonators, where modest, localized actuation is sufficient.

Although the electrode configuration used in this study was not fully optimized, it was possible to achieve vibration amplitudes sufficient for non-contact mode operation. Future efforts will focus on refining the electrode design—particularly through the use of finger electrodes—in order to enhance field distribution and improve actuation efficiency. In parallel, more extensive material characterization and further investigation of the electromechanical behaviour of ZnO nanoparticle layers will be conducted to support their potential application in MEMS.

## Figures and Tables

**Table 1:** Resonance frequency values and the related deflection maxima for each probe at various measurement conditions.

Probe No.	Without ZnO—NP		With ZnO—NP			
	Brownian		Brownian		Applied Generator Stimulation	
	f_res_ [kHz]	Deflection Amplitude [pm]	f_res_ [kHz]	Deflection Amplitude [pm]	f_res_ [kHz]	Deflection Amplitude [pm]
1	93.67188	0.99649	93.98438	0.81215	94.03125	1182.139
4	77.875	0.65546	77.82813	0.75621	77.89063	381.2466
5	100.125	0.48125	100.2188	0.77490	100.3125	1036.352
6	86.6875	0.60393	86.71875	0.67285	86.76563	828.1655

## Data Availability

Data is contained within the article.
